# A low-cost and open-source platform for automated imaging

**DOI:** 10.1186/s13007-019-0392-1

**Published:** 2019-01-28

**Authors:** Max R. Lien, Richard J. Barker, Zhiwei Ye, Matthew H. Westphall, Ruohan Gao, Aditya Singh, Simon Gilroy, Philip A. Townsend

**Affiliations:** 1 Russell Labs, University of Wisconsin-Madison, 1630 Linden Drive, Madison, WI 53706 USA; 2 Birge Hall, University of Wisconsin-Madison, 430 Lincoln Drive, Madison, WI 53706 USA; 3 Frazier Rogers Hall, 1741 Museum Road, Gainesville, FL 32611 USA

**Keywords:** Automated, Hyperspectral imaging, Open-source, Imaging spectroscopy, Non-invasive, Reflectance, *Arabidopsis*, Low-cost

## Abstract

**Background:**

Remote monitoring of plants using hyperspectral imaging has become an important tool for the study of plant growth, development, and physiology. Many applications are oriented towards use in field environments to enable non-destructive analysis of crop responses due to factors such as drought, nutrient deficiency, and disease, e.g., using tram, drone, or airplane mounted instruments. The field setting introduces a wide range of uncontrolled environmental variables that make validation and interpretation of spectral responses challenging, and as such lab- and greenhouse-deployed systems for plant studies and phenotyping are of increasing interest. In this study, we have designed and developed an open-source, hyperspectral reflectance-based imaging system for lab-based plant experiments: the HyperScanner. The reliability and accuracy of HyperScanner were validated using drought and salt stress experiments with *Arabidopsis thaliana*.

**Results:**

A robust, scalable, and reliable system was created. The system was built using open-sourced parts, and all custom parts, operational methods, and data have been made publicly available in order to maintain the open-source aim of HyperScanner. The gathered reflectance images showed changes in narrowband red and infrared reflectance spectra for each of the stress tests that was evident prior to other visual physiological responses and exhibited congruence with measurements using full-range contact spectrometers.

**Conclusions:**

HyperScanner offers the potential for reliable and inexpensive laboratory hyperspectral imaging systems. HyperScanner was able to quickly collect accurate reflectance curves on a variety of plant stress experiments. The resulting images showed spectral differences in plants shortly after application of a treatment but before visual manifestation. HyperScanner increases the capacity for spectroscopic and imaging-based analytical tools by providing more access to hyperspectral analyses in the laboratory setting.

**Electronic supplementary material:**

The online version of this article (10.1186/s13007-019-0392-1) contains supplementary material, which is available to authorized users.

## Background

Remote monitoring of plant physiology and biochemistry holds enormous potential for understanding plant growth and development in settings ranging from the laboratory to the field [1]. Reflectance spectroscopy is rapidly emerging as a highly effective and practical approach for the rapid, non-destructive estimation of a wide variety of chemical, biophysical, and metabolic plant traits in living tissue [2]. The technique uses variations in leaf optical properties that arise from the interaction of light and chemical bonds [3, 4]. For example, measurement of absorbance and reflectance features in the visible spectrum and out into the infrared (~ 400 to 2500 nm) have been used to directly estimate foliar structure, plant chemical composition, water content, and metabolic status [5–9]. Some spectral features are known to be associated with specific chemical or stress responses, such as the detection of plant physiological stress using the photochemical reflectance index [10–13]. These spectra remain a rich resource information yet to be mined for plant studies and phenotyping, with the potential for many additional features of plant physiology and chemistry to be extracted [14, 15].

While such spectroscopic imaging techniques offer huge potential, they also raise many practical issues that currently limit applications [1]. For example, scheduling collections for multiple spectroscopic measurements across many samples and over many time points is often logistically difficult and even prohibitively time consuming, especially in the field where variable light conditions affect measurements [16]. These become important issues for the analysis of plant responses which tend to change rapidly in response to environmental or biotic stressors (requiring time-course data collection) and also to vary widely between different plant species or genotypes (requiring sampling from many individuals). To alleviate these setbacks, researchers and private companies have developed machines to automate most if not all of the imaging process [17–22].

The widest use of hyperspectral imaging is from sensors mounted on drones or small aircraft [23, 24]. Recently, stationary machines have also been created to scan non-moving fields or targets [1, 17, 25, 26]. In these machines, the imaging instrument is typically mounted to an optimal point and a motor-driven axis moves the plants that need to be scanned, or vice versa. The machine is controlled by software that accepts user input such as positional and instrument control commands. Although these machines can produce highly informative data from multiple samplings due to the non-invasive nature of hyperspectral imaging, there are significant drawbacks that have severely limited their accessibility to many plant researchers [27].

The main constraints with stationary imaging machines are high cost, often complex construction, and relatively large size, which all impact application in the laboratory setting [28]. For example, the LemnaTec Gmbh Lab Scanalyzer has a robust design and features a flexible arsenal of sensors, but its cost is prohibitive to most researchers [29]. Similarly, the Field Scanalyzer is suitable for field-based research, but it is even more expensive, requires a team of people to build, and can only be used in a large crop setting [18]. Custom machines have been built by researchers to provide equivalent non-invasive analysis, but a lack of information and technical details make these platforms relatively inaccessible and difficult to reproduce by most groups. In addition, differences in design for each platform mean that comparisons of results between studies can be harder to make and/or reproduce [6, 30–33].

However, with the advent of open-sourced, or so-called “maker” electronics and parts, researchers can now build these types of machines more easily. High quality, inexpensive, and well-documented tools are becoming increasingly available. Low-cost electronics and customizable materials (such as 3D printed parts) have given rise to a unique set of novel lab hardware [34, 35]. In addition, the nature of open-source materials makes sharing these new inventions easier as well [36, 37].

**Fig. 1:**
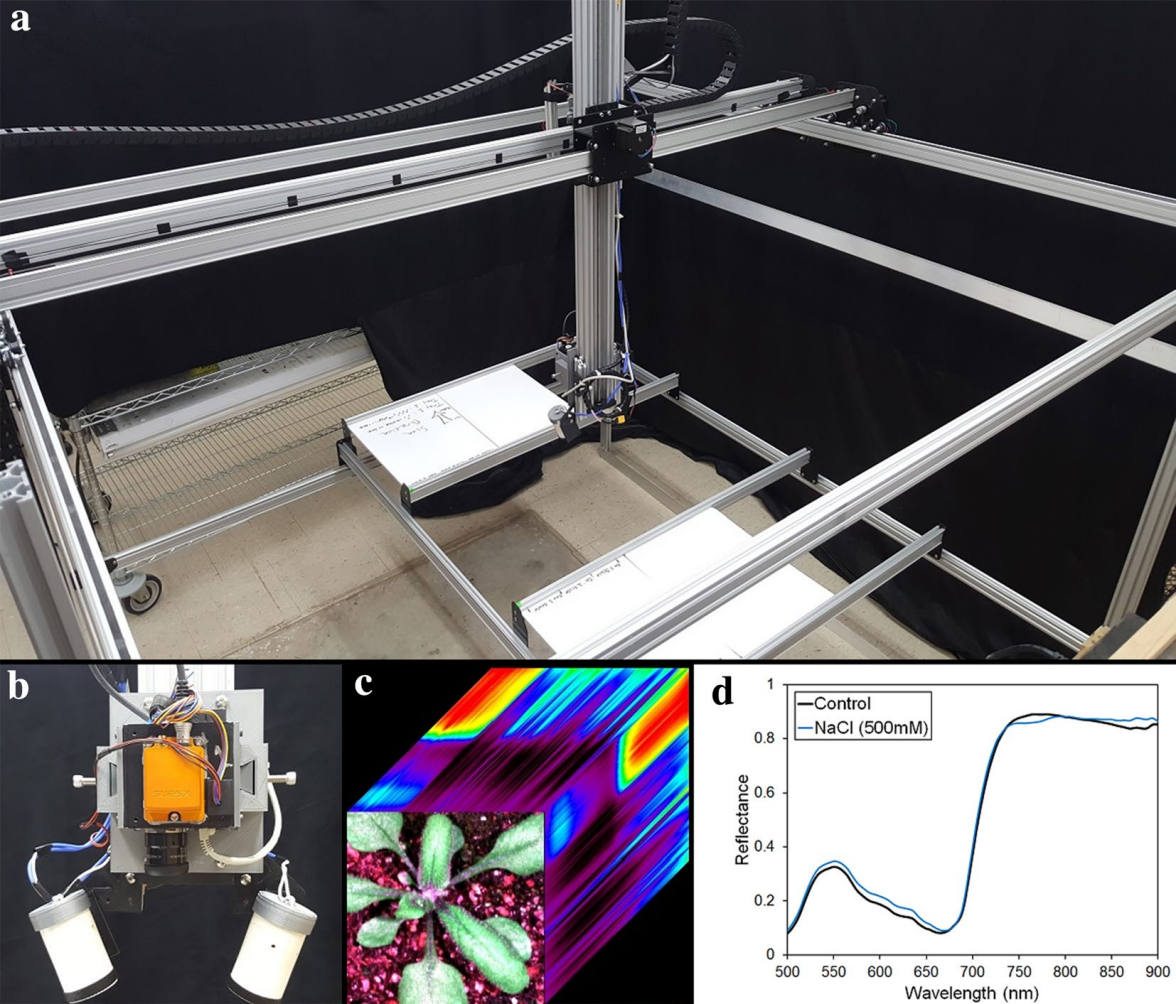
**a** Photo of the HyperScanner. **b** Modified mobile scanning head for a flat leaf experiment, equipped with the Headwall Photonics Nano Hyperspec. **c** Red–green–blue image of an *Arabidopsis* plant with a visual representation of hyperspectral data. **d** Visible and near infrared spectra of control and saline treatment *Arabidopsis* 1 day after the stress point

In this study, we have created HyperScanner: a non-invasive, lab-based system for hyperspectral imaging (Fig. 1a). Although most commercial spectrometers are not open-sourced, the HyperScanner platform itself is based entirely on other open-sourced systems and products that are also affordable. We combined an already existing open-source Computer Numerical Control (CNC) machine, the X-Carve, with custom software to create HyperScanner [38]. If one already has a preferred imaging instrument, the cost of the HyperScanner platform (not including the imaging spectrometer) totals less than 3000 USD.

HyperScanner’s hardware and software are designed with the flexibility to tailor it to specific experiment protocols with minimal commitment of effort and time. The large scanning area allows for many plant samples, with a current capacity of about ~ 20 standard seed trays, to be studied simultaneously. A scan of two trays each containing 18 *Arabidopsis thaliana* plants takes approximately 5 min. In addition to HyperScanner’s versatility, the design is fully modular: any part can be reengineered for a different sensor or experiment [39, 40]. For example, the dimensions of the instrument mount can be changed and 3D printed again to house a different instrument (Fig. 1b). Presently, the system is equipped with a Headwall Photonics (Bolton, MA, USA) Nano Hyperspec (Nano) Visible and Near Infrared (VNIR, 400–1000 nm) detector but has the flexibility to integrate other imaging modalities to provide an even deeper set of structural and chemical data to monitor plant performance (Fig. 1b, c). Further, the aim of this project was not only to create a low-cost and lab-based imaging machine, but also to provide documentation on its operation and construction with the goal of making this type of machine more accessible to plant researchers. Details regarding technical information, construction, and software are discussed in the methods section.

We validated HyperScanner for plant research by imaging *Arabidopsis* plants experiencing drought or saline stress, as both are easily imposed and controlled environmental stresses [41]. Along with the readily available tools to quantify plant health by means of Red–Green–Blue (RGB) photography, *Arabidopsis* was chosen as the test subject because of the extensive literature characterizing its responses to a wide range of environmental conditions [42, 43]. This broad background of knowledge allows us to place insights from HyperScanner’s spectral data into the broader context of physiological, biochemical, and molecular changes already characterized under those conditions. The HyperScanner was able to identify spectral shifts in the plant before any physiological harm could be detected using RGB photography [44] (Fig. 1d). If applied in the field, HyperScanner would allow the horticulturist or agronomist to amend the environment or use stress resistant varieties to ensure robust crop yields [45, 46].

## Results

HyperScanner proved to be a consistent and reliable tool that is able to collect reflectance data. The construction of HyperScanner consisted of low-cost and open-source materials, which resulted in a modular design. This approach allows the system to be modified and customized to support many different kinds of sensors and experiments. In our case, we optimized the position of the light mounts so that they provided effective lighting for an *Arabidopsis* experiment (Fig. 1b). Our custom software ensured that the operation of HyperScanner was not only reliable but also intuitive for the user.

**Fig. 2:**
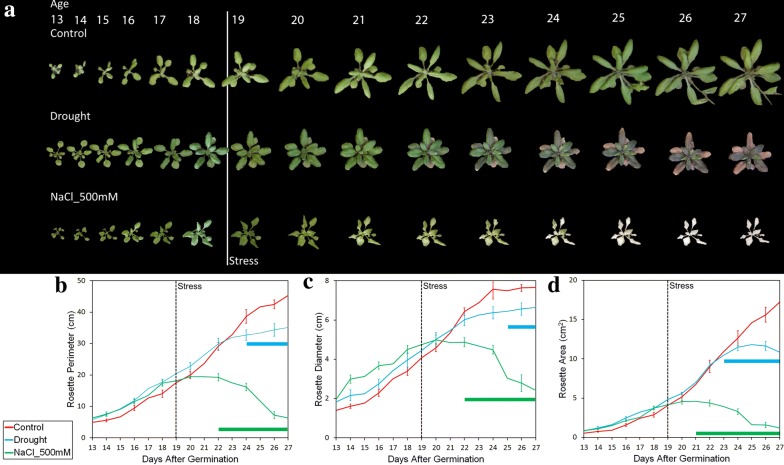
**a** Representative images of wild type Col-0 *Arabidopsis* responding to drought and 500 mM NaCl stress. Plants were grown for 19 days, before applying stress treatments. Images were analyzed using the Phenotiki image analysis software: **b** Rosette perimeter, **c** rosette diameter, and **d** rosette area (**b**–**d** mean ± SE, n = 18 replicates). Bars represent points significantly different from control, t-test, p < 0.05

We grew the plants for 19 days in preparation for the stress period and hyperspectral analysis and used daily time-lapse photography with an 8MP Raspberry Pi Camera (RGB imaging; Raspberry Pi camera V2; Adafruit, New York, NY, USA). Differences in plant morphology were observed by performing this time-lapse photography of plant growth and applying the Phenotiki analysis package [42] (Fig. 2a).

On day 19, water was added to the control plants, the salt stress sample was given 2 L of 500 mM NaCl solution as an osmotic and ionic stress, and the drought sample was allowed to dry out by withholding watering from this time point onwards. The plants’ growth environment (weight, moisture level and temperature) was monitored from day 13 until the final scan, and the data is available in Additional file 1.

Analysis of plant morphology is presented in Fig. 2b–d. The investigation of morphological traits showed a reduction in growth rates of the drought samples and a gradual decline of plant size in the saline group (that correlates with the bleaching of the leaves) (Fig. 2a). Visual indicators of stress response represented by trends in reduction in rosette diameter, perimeter, and area were detectable 1–2 days after the stress point (Fig. 2a). Statistical analysis of these parameters showed a significant difference 1–2 days after the saline treatment and 4–6 days after the drought period began (Fig. 2b–d). On day 23, significant effects from saline stress on all traits occurred but only a significant effect on leaf area due to drought could be seen. Drought effects on leaf diameter and perimeter were significant on days 24 and 25, respectively. Thus, using conventional morphometric analysis it was possible to see a difference on day 24 for the drought and day 21 for the saline stress.

**Fig. 3:**
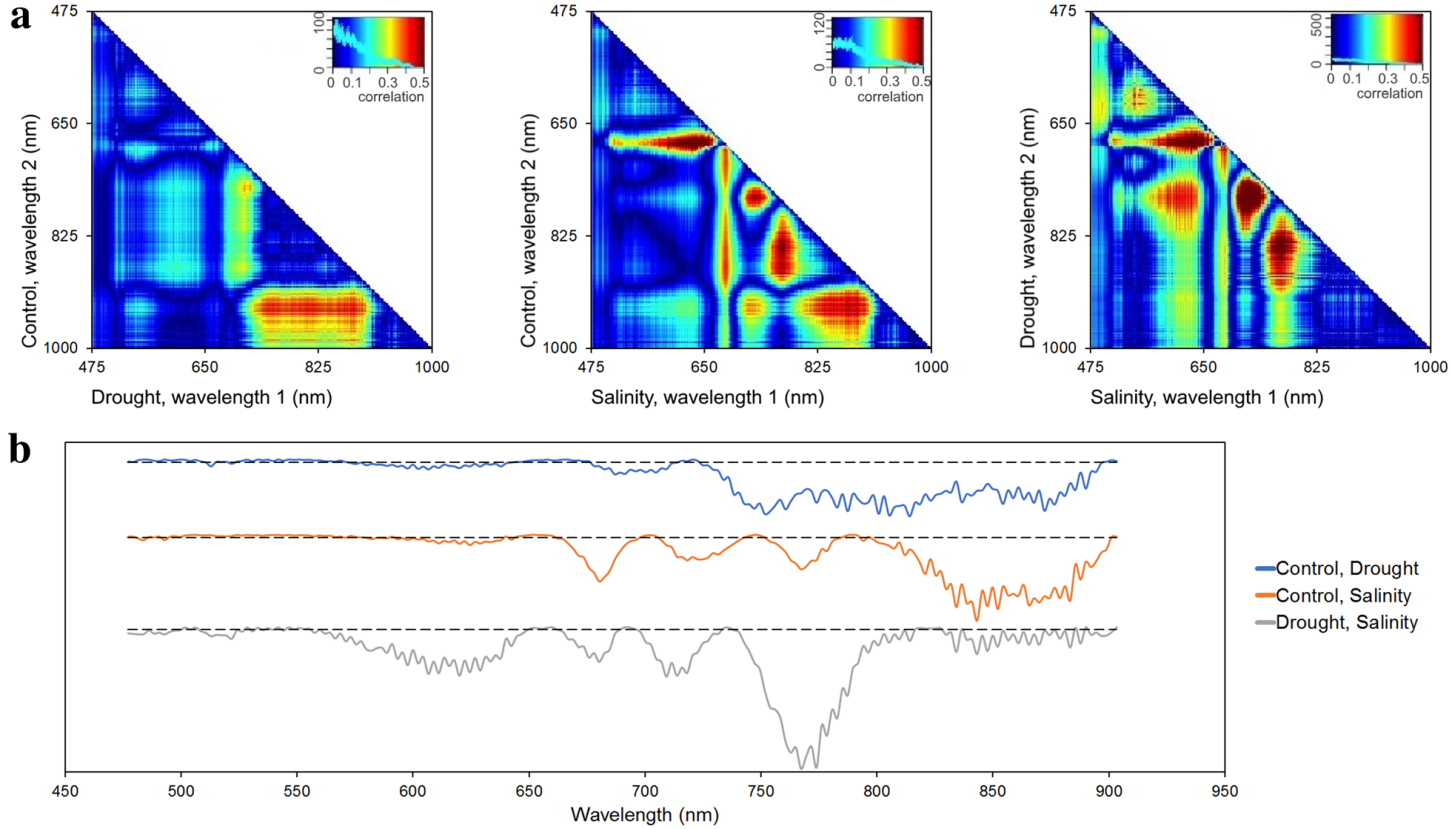
Analysis of reflectance data on day 20. **a** NDSI correlation representations between each combinations of treatments. **b** Wavelength by wavelength t-tests for each treatment combination. Each solid line is the log (base 10) of the resulting p values. Each dashed line corresponds to the value showing statistical significance, log_10 (p = 0.05): values below the line indicate significance

Hyperspectral scanning began on day 20 (1 day after the stress point) and continued until day 26. Radiance images were converted to absolute reflectance and vector normalized. Pixels containing plants were extracted and sample pixels (n = 2000) were used for further analysis. Reflectance curves on day 20 revealed significant contrasts between the stress and control groups and provided a rapid indication that the plants were experiencing either drought or saline stress. Reflectance data measured on day 20 are presented as Normalized Difference Spectral Index (NDSI, comparing all wavelength pairs) heatmaps and wavelength by wavelength t-tests in Fig. 3. NDSI correlation (r-value) heatmaps indicate statistical trends in all treatment combinations (Fig. 3a). The NDSI heatmaps show narrowband sensitivity to drought at ~ 700 to 900 nm, and saline addition at ranges ~ 500 to 650 nm, ~ 700 to 720 nm, and ~ 800 to 900 nm. Differences between the drought and salinity are present at ~ 580 to 650 nm and ~ 750 to 800 nm. Although each sample combination exhibited unique trends, significant effects on wavelengths in the red-edge and near-IR ranges are present in all sample groups.

Wavelength by wavelength p values from t-tests on day 20 reflectance data between treatments are presented in Fig. 3b. A log (base 10) plot of the p values illustrates wavelengths in the reflectance spectrum with the greatest significance for differentiating treatments (values below the dotted lines on Fig. 3b). The resulting p values denote the specific wavelengths that changed due to stress. Each sample combination exhibited major significance in the 650–700 nm range and wavelengths past the red edge inflection point (~ 700 nm), and minor significance in the 500–650 nm range. Drought samples exhibited significant effects broadly in the near infrared, related to leaf/plant structure. Salinity samples exhibited narrower features, particularly at red wavelengths (related to effects on chlorophyll), at the red-edge (720 nm) due to stress, and in several locations in the near infrared due to impacts on leaf structure. As well, p values between the two stress treatments reveal differences in the patterns that all plant samples experienced. Notably, the two treatments exhibited significantly different responses in the green and red wavelengths, at the red-edge (greater effects of salinity in green, red and near-infrared) and very significant differences at 770 nm. While each treatment showed significant effects relative to the control at longer near-infrared wavelengths (> 800 nm), the two treatments were less distinguishable from each other at these wavelengths, pointing to the utility of broadband versus narrow spectral data. Significant differences between all sample groups could be observed on day 20, i.e., several days before the morphological RGB analysis was able to do so.

**Fig. 4:**
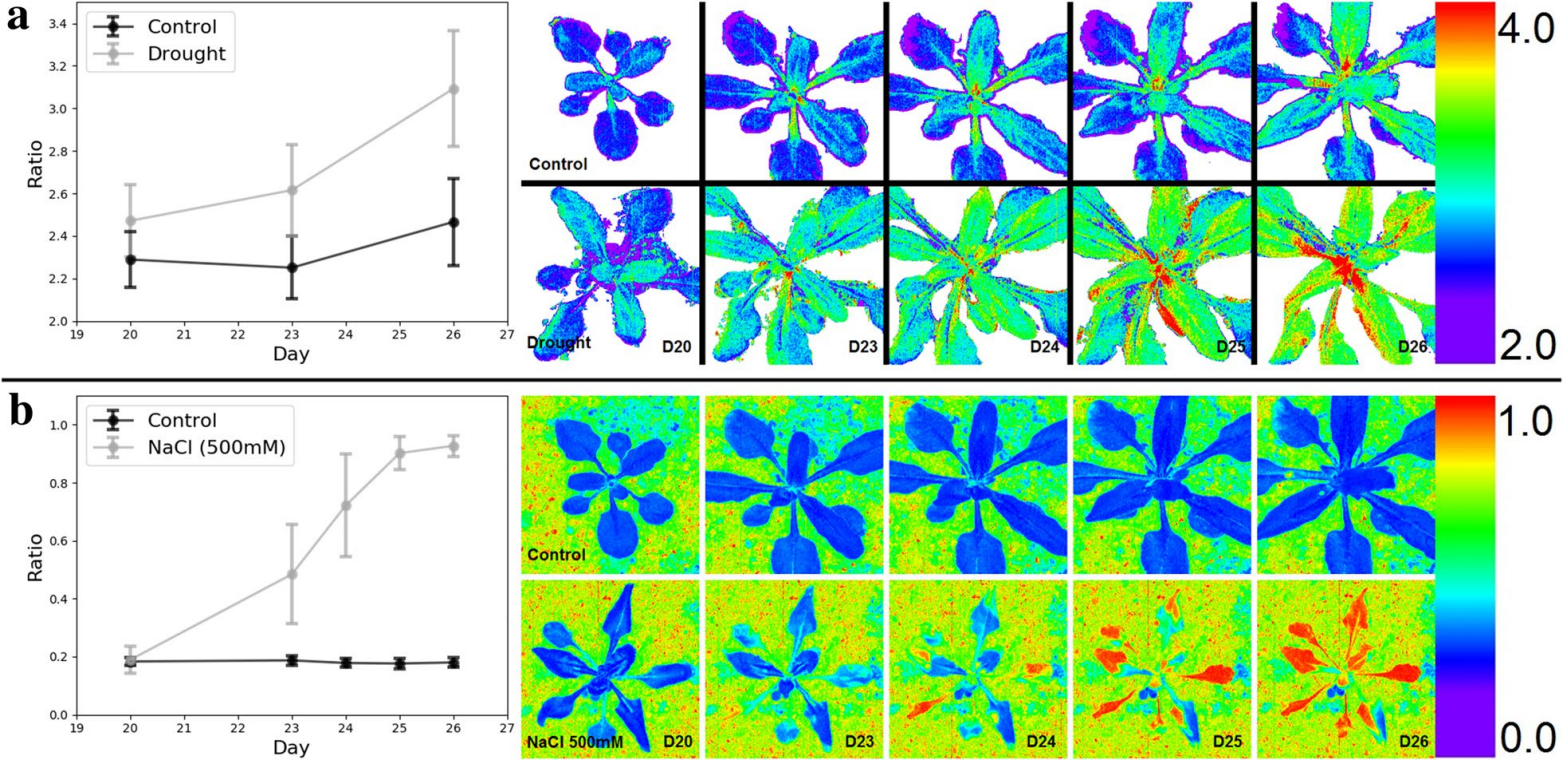
**a** Spectral response of plants due to drought stress. Leaf reflectance ratio of 782 nm and 544 nm (i.e., R782/R544) as: trend in leaf subsets (mean ± SD), time series of representative control and drought stress plants. **b** Spectral response of plants due to saline stress. Ratio-metric comparison (R676/R743) of leaf reflectance as: trend in leaf subsets (mean ± SD), time series of representative control and drought stress plants. The wavelengths chosen for ratios are confirmed by the significant relationships shown in Fig. 3

Figure 4 presents trends in the stress plants from day 20 to day 26 using reflectance ratios derived from the hyperspectral imagery, based on significant wavelengths identified in Fig. 3. This enables visualization of changes in plant stress as it progresses by date. Ratios specific to the stress type were calculated with representative wavelengths, 782 nm/544 nm to compare drought stress with the control and 676 nm/743 nm to compare the salinity stress with the control. The calculated ratios were interpolated into each pixel of representative plant images on days 20, 23, 24, 25, and 26. Differences with the control are seen on day 20 in each stress type. Generally, ratios increased over time in stress samples and little to no change was seen in the control. In contrast to the day 20 analysis (Fig. 3), this method allowed us to observe spectral shifts in the spatial domain as well as across a period of time.

To assess the radiometric fidelity of the Nano measurements, we compared reflectance from a flat leaf of a control plant made with an Analytical Spectral Devices full-range contact spectrometer and calibrated light source (ASD; FS3 350-2500; Analytical Spectral Devices, Boulder, CO, USA) with the Nano image of the same plant. The comparison demonstrated the congruence between measurements from the two types of instruments (Additional file 2), indicating that the measurement setup for the HyperScanner with the Nano (i.e., light source, calibration panels) is sufficient to match leaf level reflectance from a higher Signal-to-Noise Ratio (SNR) instrument such as the ASD.

## Discussion

Plants experience a range of abiotic stresses in natural ecosystems, and in the context of an agroecosystem, environmental stresses can lead to reductions in growth rate and altered vegetative and reproductive development, which often plays out as being detrimental to crop yields. We mimicked environmental stresses common to agroecosystems within our controlled environment [47] to ask how well the HyperScanner could be used to rapidly monitor plant responses to these challenges. As the plants sustained the effects of drought or salinity, visual symptoms of the stress appeared (Fig. 2a). Monitoring plant growth by means of conventional RGB photography coupled to statistical analysis of the morphometric data extractable from these images allowed us to define a point when the physiological effects from the stress were detectable as being statistically significant alterations in leaf growth rates from the control. Thus, analysis of rosette diameter, perimeter, and leaf area indicated that the drought samples’ growth rates were not statistically significant until day 23, i.e., 4 days after imposition of drought by cessation of watering (Fig. 2). The addition of the 500 mM saline solution affected the plants more rapidly than the drought stress. The salinity caused the plants to exhibit chlorosis of the leaves [48] and statistical significance on leaf expansion was again seen on day 22. In both treatments, statistical significance of plant growth responses to stress application was detected several days after the stress application. This delay is likely due to the limitations of visual analysis of parameters, such as growth, on detecting the earliest responses to stress that are likely to be through alterations in gene expression and plant biochemistry [49]. If a farmer or horticulturist were to be solely relying on analysis of RGB photography to assess stress conditions in the field, significant reduction in plant size and yield would be inevitable as these would be tightly linked to the changes being used to detect stress response from the imaging data.

By using hyperspectral data, the amount of information available to a researcher dramatically increases [50, 51], and so, the RGB analysis in Fig. 2 does not contain the vast amount of information that the hyperspectral imaging can potentially provide. Thus, statistical analysis from the hyperspectral data provided information on plant stress being statistically significant well before alterations in growth were detected from the RGB data. The NDSI analysis in Fig. 3a reveals effects on the plants 1 day after the stress period began. When compared to the control, the drought stress was most significant in red edge to near IR bands (~ 700 to 900 nm). The heatmap of the salt and control revealed more significant trends (both magnitude and quantity), reinforcing that the saline stress was likely affecting the plants to a greater degree (at least in terms of changes in hyperspectral signal) than the drought stress (Fig. 2). The salt and drought NDSI show that the two types of stress have comparable effects on *Arabidopsis* 1 day after stress. Red-edge and near-IR wavelengths show significance in both treatments when compared to the control, but not in their own comparison, indicating that the two samples were affected in the same range of wavelengths and to similar amounts. On the other hand, correlations in the 580–650 nm and 750–800 nm ranges are present in the third NDSI and are not seen in comparisons with the control, which suggests that the stress samples changed differently in these ranges.

Analyses of p values in Fig. 3b reveal changes along certain wavelengths in treatments and confirm our hypotheses drawn from the NDSI heatmaps in Fig. 3a. Significant p values resulting from the comparison of 580–640 nm between the two stress treatments indicate that these changes are unique to each stress type (Fig. 3b), namely through greater effects on reflectance in the saline treatment potentially due to differences in changes in relative pools of accessory (non-chlorophyll) pigments between the treatments. Similarly, wavelengths between 600 and 650 nm changed in both stresses when compared to the control, but when the stresses are compared against each other, major statistical differences are present, indicating that the effect on the (most likely) chlorophyll absorption in the red was much stronger with the saline treatment. As well, significance in the 700–800 nm range affirms that the NDSI correlations from that range are indeed unique to the stress type, with a greater impact on red-edge reflectance (an indicator of overall plant health) in the saline treatment. T-tests between the stress types allow for powerful conclusions to be made that are not available with comparisons to the control. Not only can change be seen between stresses and control groups, but the change relative to different stresses can be analyzed.

Figure 3 also suggests that certain response mechanisms were employed by the plants to each stress condition. The drought samples experienced a shift in reflectance at the ~ 520 to 530 nm wavelengths compared to the control. This corresponds to the location of the photochemical reflectance index band at 531 nm [10], which has been shown to relate strongly to plant xanthophyll cycle pigment pools that change in response to stress [11]. In contrast, the ~ 520 to 530 nm band was not significantly changed in the saline stress, which confirms that the associated physiological change was likely only experienced by the drought stress. Similarly, the saline stress saw shifts in the sub-500 nm range that were not present in the drought samples, perhaps due to effects on chlorophyll-b and carotenoids that have strong absorptance features in the blue [52]. Similar observations can be drawn on different wavelengths. In each treatment, the most significant changes are seen after 700 nm and into the near-IR range. In the 750–800 nm range, both stress sample’s reflectance shifted compared to the control, and the drought shifted more than the salinity. Analysis between the treatments reinforce this idea, as t-tests on those wavelengths resulted in the smallest p values. In addition, bands in the near-IR range were significant in both the drought and salinity samples; however, the *t* test between the two stresses shows only a small degree of significance, demonstrating that the plants’ reflectance changed in a similar manner.

Visualization of the hyperspectral imagery in Fig. 4 offers the capacity to track the progression of change using reflectance-based plant experiments. Because the response wavelength ratios can be visualized on the plant itself, spatial trends can be analyzed over time along with spectral trends. For instance, the effects on the drought ratio were initially noticeable at the base of the plant but spread through the stem and then to the leaves along the vasculature (Fig. 4a). Interestingly, the older salt stressed leaves experienced a reflectance change before the younger leaves (Fig. 4b). Such analyses are not possible if one only considers purely spectral data.

HyperScanner is easy and inexpensive to build and suitable for many varied plants and experiments. Any plant can be scanned as long as they can fit into the scan area and is not taller than the height of the instrument, although, the height of the instrument, scanning speed, and the scanning routes can be changed to accommodate different species of plants. For example, in addition to *Arabidopsis*, we have successfully used HyperScanner on much larger cotton plants.

Along with being able to support many different plants, HyperScanner can support different sensors and indeed, expansion to incorporate multiple parallel imaging modalities is a core concept for the HyperScanner. Thus, the 80/20 rail system combined with printing custom mounts allows for the rapid integration of new sensors. In this study, only the Nano was mounted as a detector in order to efficiently test the feasibility of HyperScanner. One notable current limitation is that the Nano is operated through the manufacturer’s software. This makes starting the scans slightly more cumbersome. The full integration of the Nano into our software will increase the quality of operation.

We are also currently implementing other sensors, including a laser rangefinder to detect and compensate for different plant heights [27]. *Arabidopsis* is very flat and not variable enough in height for a rangefinder to have been relevant for this study, but this will be an important addition for larger, more 3-dimensional plants. A parallel thermal imaging system will allow for the assessment of changes in the critical parameters of transpiration and photosynthetic capacity and a laser scanner will further enable the assessment of changes in foliar structure and biomass across future experiments [6, 14, 27, 53]. The integration of these imaging systems will be documented in future work using the HyperScanner.

Perhaps the greatest potential for HyperScanner is in full automation of control and analysis [54]. The control system is currently being transformed from open-loop to closed-loop (i.e., using internal sensors and feedback) control. In addition, an exciting area for future development is to incorporate neural networks for plant classification [55]. The combination of HyperScanner and neural networks will allow for even more rapid acquisition and classification of reflectance data [28, 56]. Automated stress detection via a neural network could allow a researcher to maintain healthy samples with minimal interference. Indeed, the complete automation of the HyperScanner will result in an extremely efficient system [25].

## Conclusion

The HyperScanner system was designed to collect hyperspectral data with minimal human effort while keeping the system accessible to researchers, affordable, and available for the addition of more instruments. The software, data, and other relevant files are publicly available within the article. In multiple experiments, we were able to measure absolute reflectance in *Arabidopsis* stress experiments. The data from HyperScanner showed spectral differences at an earlier point during the stress than visual observations and identified differences in stress responses between two treatments. HyperScanner can be used for improved detection of plant stress and holds a high potential to be a commonplace method for studying plants in many research settings.

## Methods

### *Arabidopsis* growth environment

A controlled growth chamber was used to grow plants adjacent to the HyperScanner. Six 1020 seed trays (Greenhouse MegaStore, Danville, IL, USA) each with 18 *Arabidopsis Col*-*0* plants growing in potting soil were maintained at 22 °C with an 18:6 h day/night cycle (100 µmol/m^2^/s^−1^, from 4 foot fluorescent lights). Pot weights and RGB pictures of the plants were taken daily. Plant phenotypic response was analyzed from Raspberry Pi camera images using Phenotiki [42]. Hyperspectral scans were taken each day for 7 days starting at 20 days post germination. Two trays were used as control samples and were kept with constant water availability by adding 500 mL every day. The remaining four trays were split into 2 treatments. Two trays were not watered from day 19 onwards to impose drought stress. The two other trays were used for salt stress experiment by adding 2 L of 500 mM NaCl. To mitigate a possible border effect, edge plants should be excluded from data analysis and uniform lighting across the plant tray should be ensured.

### Overview of CNC and HyperScanner

HyperScanner is based on CNC technology [57]. CNC is an automation of machine tools that utilizes computer control, smart sensors, and stepper motors. In CNC, the control computer executes sequential commands which are calculated based on user inputs and the machine’s physical properties; smart sensors provide necessary information used to execute the computer’s control algorithms. Stepper motors control the physical position of the tools. The computer-based control of CNC allows processes to be predetermined and also enables the recalibration of computer commands based on external changes. This digitization results in an automated and high-precision system.

HyperScanner can precisely and accurately move to a point (X, Y, Z) with a user-chosen speed. The detector (in our case, the Nano Hyperspec line scanner, see below) is mounted to a central point which can move along the X, Y, and Z axes. The central point moves over the scan area underneath which facilitates the scanning of the plants. Our software gives the user the ability to intuitively control the movement of the machine in real time, create pre-planned scanning routes, and execute those routes.

### Nano Hyperspec Imaging Spectrometer

The Nano is a line scanner (also known as pushbroom scanner) designed for the VNIR range (400–1000 nm) [58]. It consists of 640 spatial bands (pixels) and 270 spectral bands. The spectral bands are spaced at 2.2 nm/pixel. The Nano weighs 0.544 kg and has built-in mounting points, making it extremely suitable for the HyperScanner.

### Technical specifications of the HyperScanner

HyperScanner was built to achieve a large scan area, variable speed, and precise movement control. HyperScanner features a scan area of 2.1 m^2^. Due to the constraints of imposed by the operating speed of the Nano, HyperScanner runs at a scanning speed of 1 cm/s; however, this may vary when using other detectors, and so the speed can be adjusted within the HyperScanner interface. Additionally, as the Nano is a line scanner, the length of the scan line is proportional to the height of the Z axis. The height can be optimized to a line that covers the sample dimensions. For instance, a line of ~ 8 cm was used in this experiment to cover the length of each pot. Table 1 lists the relevant technical values.

**Table 1:** HyperScanner’s technical specifications

Specification	S.I.
Dimensions (L × W × H)	1.8 m × 1.8 m × 1.8 m
Scan/workable area	2.1 m^2^
X axis travelling capacity	1.35 m
Y axis travelling capacity	1.55 m
Z axis travelling capacity	85 cm
Positioning accuracy	± 0.10 cm
Optimal scanning speed	1 cm/s
Maximum safe travel speed	8 cm/s
Line scan length (mounted Nano)	0.53–45.55 cm

### Construction of the HyperScanner

**Fig. 5:**
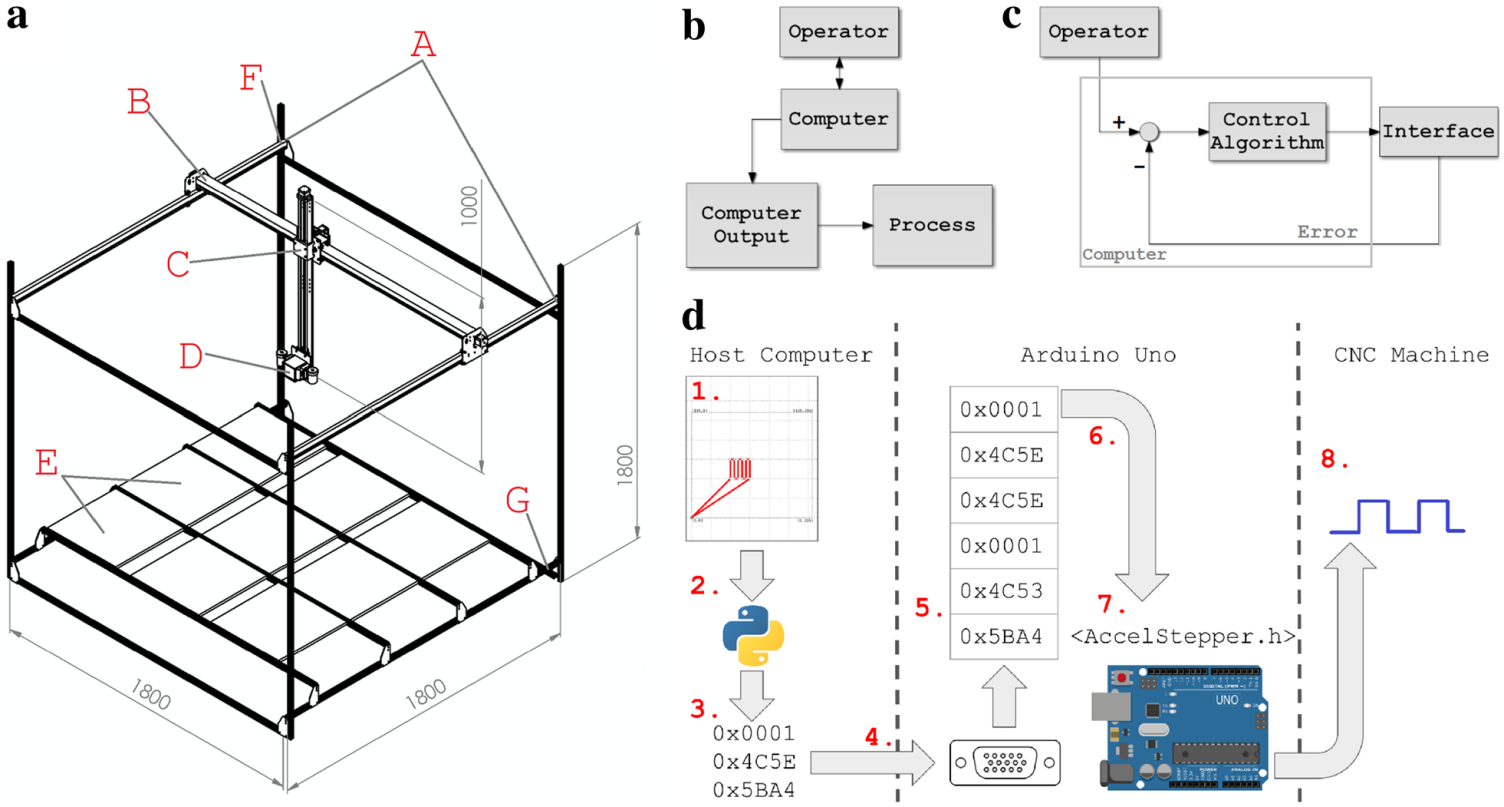
**a** Isometric view of the HyperScanner: A, Y axis rails; B, X axis rail; C, Z axis linear actuator; D, Nano Hyperspec; E, plant trays; F, top section; G, bottom section. Diagrams representing the control flow of the HyperScanner and CNC machines: **b** open-loop control and **c** closed-loop control. **d** Data flow from the user to HyperScanner: (1) user draws a scanning route in embedded web form. (2) Path waypoints are sent as ASCII to Python 3 script via CEFPython. (3) Python 3 script converts ASCII coordinates to binary tuples for Arduino instruction set. (4) Binary tuples are transmitted from host computer to Arduino via UART connection. (5) Instructions are enqueued in FIFO in Arduino memory as they arrive from UART. (6) Instructions are dequeued as previous instruction completes, and AccelStepper functions are invoked. (7) AccelStepper library computes signal required to move stepper motors. (8) Signal sent to gShield over the Arduino GPIO ports

HyperScanner was built using the 80/20 aluminum rail system, and the construction is based on X-Carve’s existing system [38]. Although detailed building instructions can be found on X-Carve’s website, many adjustments were made for our purposes [40, 59]. 3D models of HyperScanner are presented in Fig. 5a and Additional file 3. Four 1.8 m rails are vertically placed at each corner of the machine to support eight additional 1.8 m rails which are used to create two 1.8 m by 1.8 m horizontal sections. The bottom section supports removable platforms on which plant trays or pots can be scanned. The top section supports the stepper motor movement system. Two stepper motors are mounted to metal side plates that are attached to the top section support rails (Fig. 5a, Additional file 3). The side plates attached with motors are mounted with wheels that allow them to slide along two of the top section support rails (Y axis). The X axis rail is between the side plates. A metal gantry with wheels moves across this rail and also supports the linear actuator (Z axis). The X and Y axes operate with a belt drive and the Z axis operates with a worm drive. The Nano and lights are mounted at the bottom of the linear actuator by using custom-made mounts. Custom made mounts and additional support brackets were designed within SolidWorks, and each mount was specifically designed to match the existing hardware. The mounts were printed using an open-source 3D printer with PLA filament (LulzBot TAZ 5; Aleph Objects, Inc., Loveland, CO, USA). All SolidWorks files are available for download at (10.7910/DVN/9DLR7S). The bill of materials (Additional file 4) lists all materials necessary to the construction of the HyperScanner. Necessary tools are not listed.

### Control and wiring

The control algorithms that most CNC machines implement can be classified into two categories: open-loop control and closed-loop (feedback) control. In open-loop control, the process is linear, meaning that the computer simply accepts inputs from the operator and outputs a signal to control the system. Figure 5b shows the fundamental open-loop control design [57]. In closed-loop control, sensors are placed in the system to feed back information, which enables a higher degree of automation (Fig. 5c). HyperScanner is open-loop controlled. HyperScanner consists of four stepper motors and one linear actuator. The X axis is controlled by two motors; the Y axis is controlled by one motor; the Z axis is controlled by one motor and a linear actuator that allows for precise vertical positioning. Although open-loop control is currently implemented in HyperScanner, we intend to integrate feedback control to achieve a more robust and fully automated control mechanism.

HyperScanner’s basic wiring topology is included in Additional file 5. The computer accepts user input and transcribes it to stepper motor movements, which follows the same control structure in Fig. 5b. User input is translated from the computer to the Arduino and then to the gShield through custom software. The gShield is a stepper motor driver: once the gShield receives correctly notated commands, the appropriate level of power is driven to the motors. The Arduino is powered by the computer through a Universal Serial Bus (USB) connection that also doubles as the serial connection. The stepper motors, gShield, Nano, and lights are powered by a digital-control direct current power supply. A dedicated power source has not been implemented, as the weight and power load on the linear actuator is constantly changing due to the addition of various devices. Thus, the power supply needs to be constantly adjusted.

### Lighting

Custom light mounts were 3D printed along with the Nano mount. The mounts are fitted to 20 W halogen bulbs (MR11; Simba Lighting, Torrance, CA), and the bulbs are powered close to the maximum power rating. The lights are mounted in parallel to the Nano’s scan line so maximum even lighting is achieved. The mounts are connected to the end of the Z axis so that the lighting environment does not change while the scanner is moving. Additionally, the mounts are connected to 80/20 rails that slide along the Z axis linear actuator so that their height from the samples can be adjusted.

Due to the indoor setting, artificial lighting was one of the most important considerations when building HyperScanner. The lighting and light mounts were reiteratively modified until a desirable configuration was achieved, resulting in even and consistent illumination throughout each of the scans. The interchangeability and adjustability of the lights’ power and position made this an easy task.

### Software

A set of software tools were developed to support the operation of the HyperScanner, both for direct interfacing with the machine’s hardware and for higher-level user functions. These tools, named Ardupy, have been made publicly available on the University of Wisconsin EnSpec organization’s Github page (https://github.com/EnSpec/Plant_CNC_Controller) as well as on Zenodo (10.5281/zenodo.1406721) [60]. Ardupy manages the conversion of movement instructions received over the Uno’s USB serial port into appropriate gShield instructions. Direct interfacing with the gShield is handled by the Arduino AccelStepper library [61]. AccelStepper manages the General-Purpose Input/Output (GPIO) outputs that are required to move a stepper motor to an absolute position with a given speed. User control of the AccelStepper library is achieved through a custom instruction set established between a host computer and the Arduino over a Universal Asynchronous Receiver/Transmitter (UART) serial connection. The instructions are encoded as 3-tuples of 32-bit integers that specify AccelStepper values and parameters. A full description of the instruction set is provided in Table 2. To prevent incoming instructions from interrupting the execution of previously received ones, instructions are enqueued in a First-In, First-Out (FIFO) as they are received via UART and dequeued when a previous instruction completes. In the current iteration of the software, this FIFO is implemented with the Arduino QueueList library [62]. A diagram of the data flow between the host computer and CNC machine is provided in Fig. 5d.

**Table 2:** Instruction set supported by Arduino software

Instruction	Byte 0–4	Byte 4–8	Byte 8–12
Move to X/Y axes to absolute position	1	X position in steps	Y position in steps
Set delay between each movement	2	Delay in milliseconds	0
Move X/Y axes to relative position	3	Change in X position in steps	Change in Y position in steps
Move Z axis to absolute position	4	Z position in steps	0
Move Z axis to relative position	5	Change in Z position in steps	0
Set movement speed	6	Speed in steps per second	0

In addition, the Graphical User Interface (GUI) Ardupy-GUI, was developed to facilitate the creation of scanning paths. Ardupy-GUI enables an intuitive approach to scanning. Rather than only entering values via a console, Ardupy-GUI consists of two user-friendly menus: a control panel that provides direct access to each of the instructions above; a route plotter that allows the user to draw scanning paths via click-and-drag or keyboard inputs (Additional file 6). The route plotter generates sequences of instructions based on the created paths. CEFPython, a set of Python 3 bindings for the Chromium Embedded Framework, was selected as the backend for the GUI, as it allows fast development of cross-platform graphical applications [63, 64]. CEFPython provides an interface between an embedded instance of a web browser which handles the display of the GUI and a Python 3 script which handles communication between the host computer and HyperScanner’s Arduino. The JavaScript library jQuery is used to bind user actions in the GUI to function calls in the Python 3 script that backs the GUI [65]. The route drawing tool is implemented in d3.js, a library which provides efficient manipulation of scalable vector graphic images [66]. The backend Python 3 script generates binary instructions from the textual data entered into the GUI and transmits them to HyperScanner’s Arduino over UART via the PySerial module [67].

### Operating procedures

The operation of HyperScanner is simple, after creating scanning routes and tuning instrument parameters (Additional file 7). First, the Nano is attached to the mount and connected to the computer. Scan height, speed, and line length are calculated separately, based on the Nano’s field of view and integration time. This experiment used an integration time of 14 ms. Once the power supply and lights are turned on and Ardupy has been launched, the Nano is moved to a white panel and calibrated. A scanning route is chosen, and the plant samples are placed in the correct positions according to the selected route. After the initial setup, the Nano is set to capture, and the route is executed. One scan of two plant trays (18 plants each) takes less than five minutes. After the scan is completed, files can be transferred off of the Nano or different routes and plants can be loaded. Note that a scan of the white panel should be included for image processing. After the all the plants are scanned, the images can be processed.

One important element of operation requiring attention during the equipment setup is that the belt drives must be checked to ensure they do not need to be re-tensioned. Correct belt tension is needed for optimal movement ability of the scanner and therefore this check is important. Although, re-tensioning the belts is needed very seldom.

### Image processing

Hyperspectral scans were processed with ENVI 5.0 (Exelis Visual Information Solutions, Inc., Boulder, CO, USA). Each image scan includes a calibrated 99% reflectance spectralon panel (Labsphere, North Sutton, NH, USA), which was used to calculate reflectance and estimate the noise level of the hyperspectral image. Coefficient of Variation (CV) was used as the criteria for high SNR wavelength selection. After calculating along-track CV of the white panel image, wavelengths ranging from 477.1 to 903.42 nm, which yield low CV, are considered for further analysis. The white reference radiance spectrum was estimated for the whole image from the vertical scan line of the spectralon panel that had the maximum median radiance. Every plant pixel was used for analysis by delineating regions of interest within ENVI. The spatial and spectral edges of the hyperspectral image cube were excluded from analysis because it minimizes smile and keystone effects (e.g., cross-track variation in wavelength centers) [68]. Relative reflectance is calculated as:

$$Rfl\left[ {i, j, k} \right] = \frac{{DN_{i,j,k} }}{{DN_{white,k} }}$$

where *i* and *j* correspond to the row and column of a pixel. *k* is the wavelength of the pixel. DN_*i,j*_ is the radiance spectrum of each pixel. DN_*white*_ is the radiance spectrum of the reference white panel. The calculation is done on a wavelength-by-wavelength basis.

### NDSI

Normalized difference spectral indices were calculated for each of the Nano’s 270 spectral bands. The difference in reflectance for a pair of bands (e.g., i and j) is divided by the sum, as in the following:

$$NDSI\left[ {i, j} \right] = \frac{{band_{i} - band_{j} }}{{band_{i} + band_{j} }}$$

For each pair, indices were calculated for a sample of n = 2000 pixels. Statistical tests were then done on each NDSI combination and heatmaps were generated with the resulting statistical data.

## Additional files

**Additional file 1.** Daily tray moisture, tray weight, and room temperature measurements from the controlled *Arabidopsis* growth environment. Line plot of soil conductance percentage as a proxy for soil humidity. Area line plot showing tray weight as a proxy for water content. Line plot displaying the mean temperature (22˚C ± 2˚C).

**Additional file 2.** Reflectance curves obtained by the ASD and Nano from one control *Arabidopsis* plant.

**Additional file 3.** Three additional views of the HyperScanner.

**Additional file 4.** A bill of materials listing the supplies used to construct Hyperscanner. Does not list tools.

**Additional file 5.** A basic diagram of HyperScanner’s wiring scheme. Each arrow represents a wired connection: A, external user input; B, returned data from the Nano; C, Nano control signal; D, positional data from Ardupy; E, gShield motor driver power; F, stepper motor power; G, Nano power; H, power supply; I, Arduino Uno; J, gShield motor driver; K, stepper motors.

**Additional file 6.** Screenshots of Ardupy’s manual control and route planner menus. Users can control HyperScanner with manual control or through creating paths by click-and-dragging waypoints on the map panel.

**Additional file 7.** A flowchart describing HyperScanner’s operational procedure.

## Abbreviations

CNC: computer numerical control
Nano: Headwall Photonics Nano Hyperspec
VNIR: visible and near infrared
RGB: red–green–blue
NDSI: Normalized Difference Spectral Index
ASD: analytical spectral devices
SNR: signal-to-noise ratio
USB: universal serial bus
GPIO: general-purpose input/output
UART: universal asynchronous receiver/transmitter
FIFO: first-in, first-out
GUI: graphical user interface
CV: coefficient of variation
