# Supplementary figures and images for: A low-cost and open-source platform for automated imaging
Source: Plant Methods. 2019 Jan 28;15:6. doi: 10.1186/s13007-019-0392-1 (PMC6348682; doi:10.1186/s13007-019-0392-1)

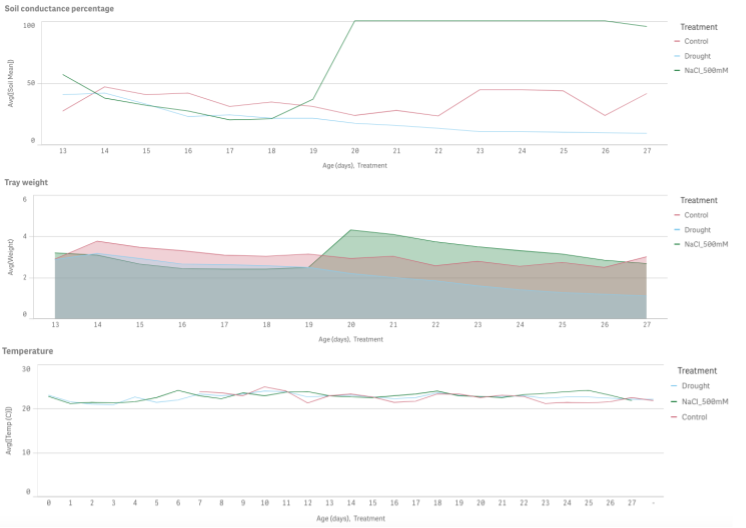

Supplement: Supplementary file 1 — Additional file 1. Daily tray moisture, tray weight, and room temperature measurements from the controlled Arabidopsis growth environment. Line plot of soil conductance percentage as a proxy for soil humidity. Area line plot showing tray weight as a proxy for water content. Line plot displaying the mean temperature (22˚C ± 2˚C). [file 13007_2019_392_MOESM1_ESM.tif]

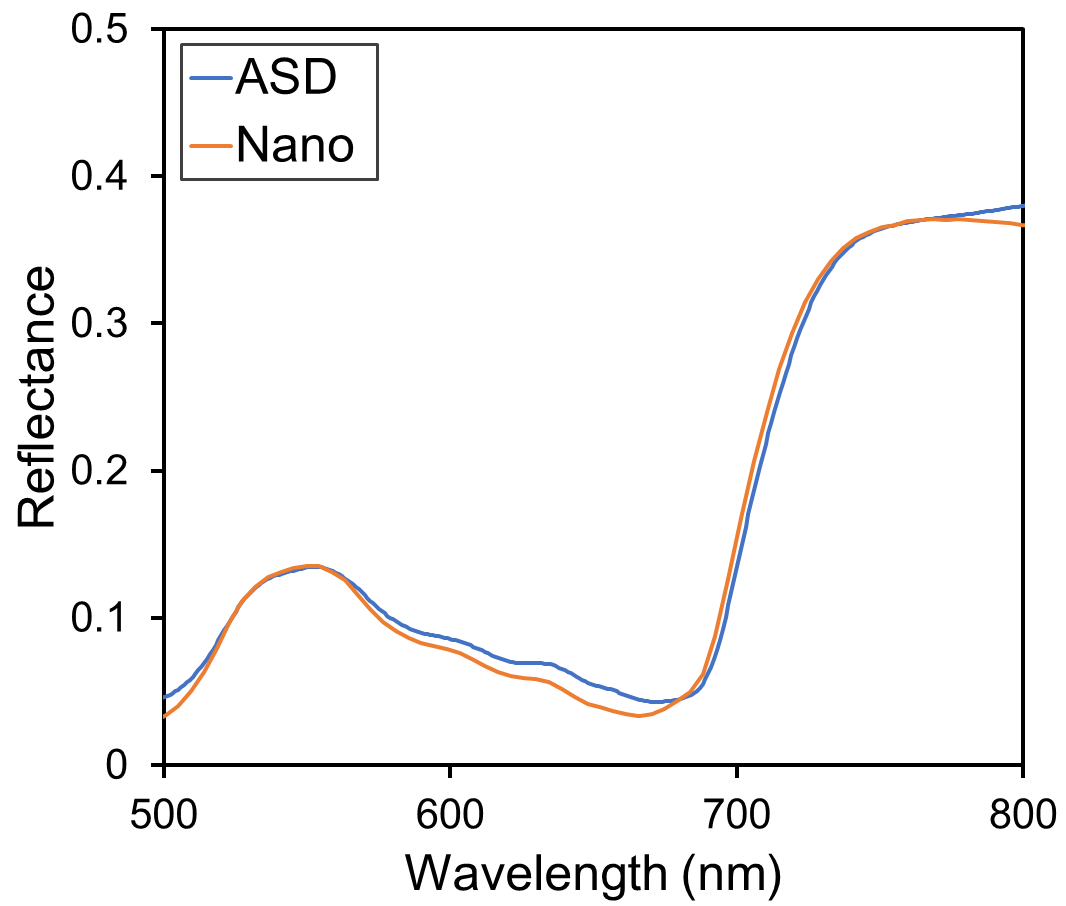

Supplement: Supplementary file 2 — Additional file 2. Reflectance curves obtained by the ASD and Nano from one control Arabidopsis plant. [file 13007_2019_392_MOESM2_ESM.tif]

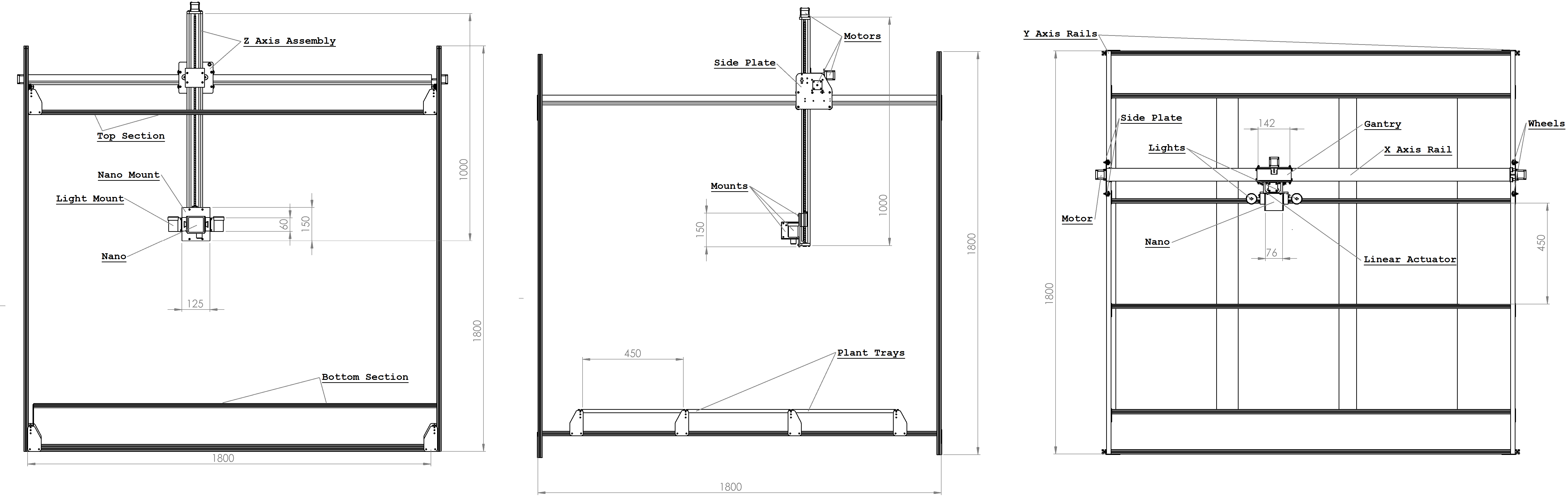

Supplement: Supplementary file 3 — Additional file 3. Three additional views of the HyperScanner. [file 13007_2019_392_MOESM3_ESM.tif]

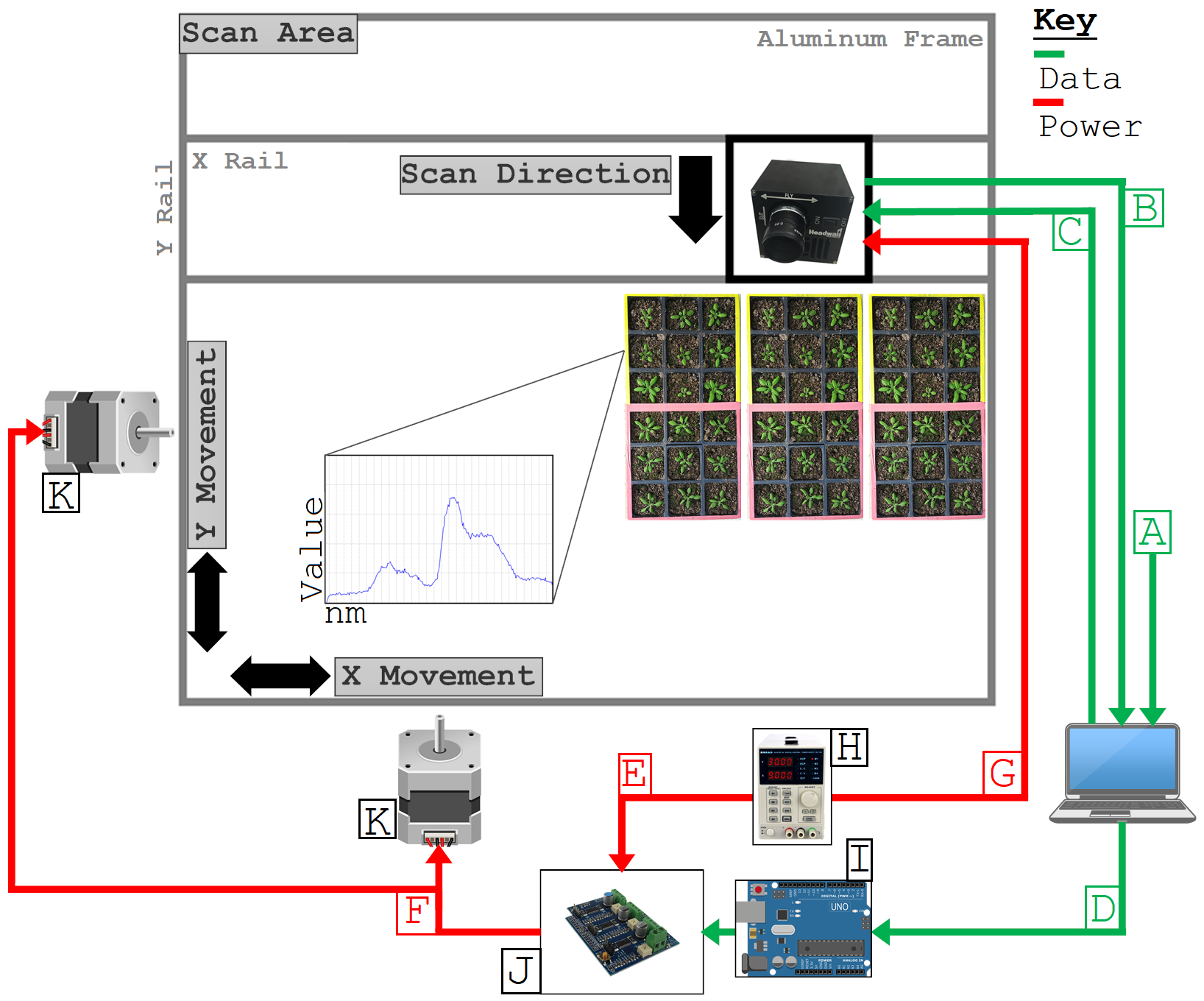

Supplement: Supplementary file 5 — Additional file 5. A basic diagram of HyperScanner’s wiring scheme. Each arrow represents a wired connection: A, external user input; B, returned data from the Nano; C, Nano control signal; D, positional data from Ardupy; E, gShield motor driver power; F, stepper motor power; G, Nano power; H, power supply; I, Arduino Uno; J, gShield motor driver; K, stepper motors. [file 13007_2019_392_MOESM5_ESM.tif]

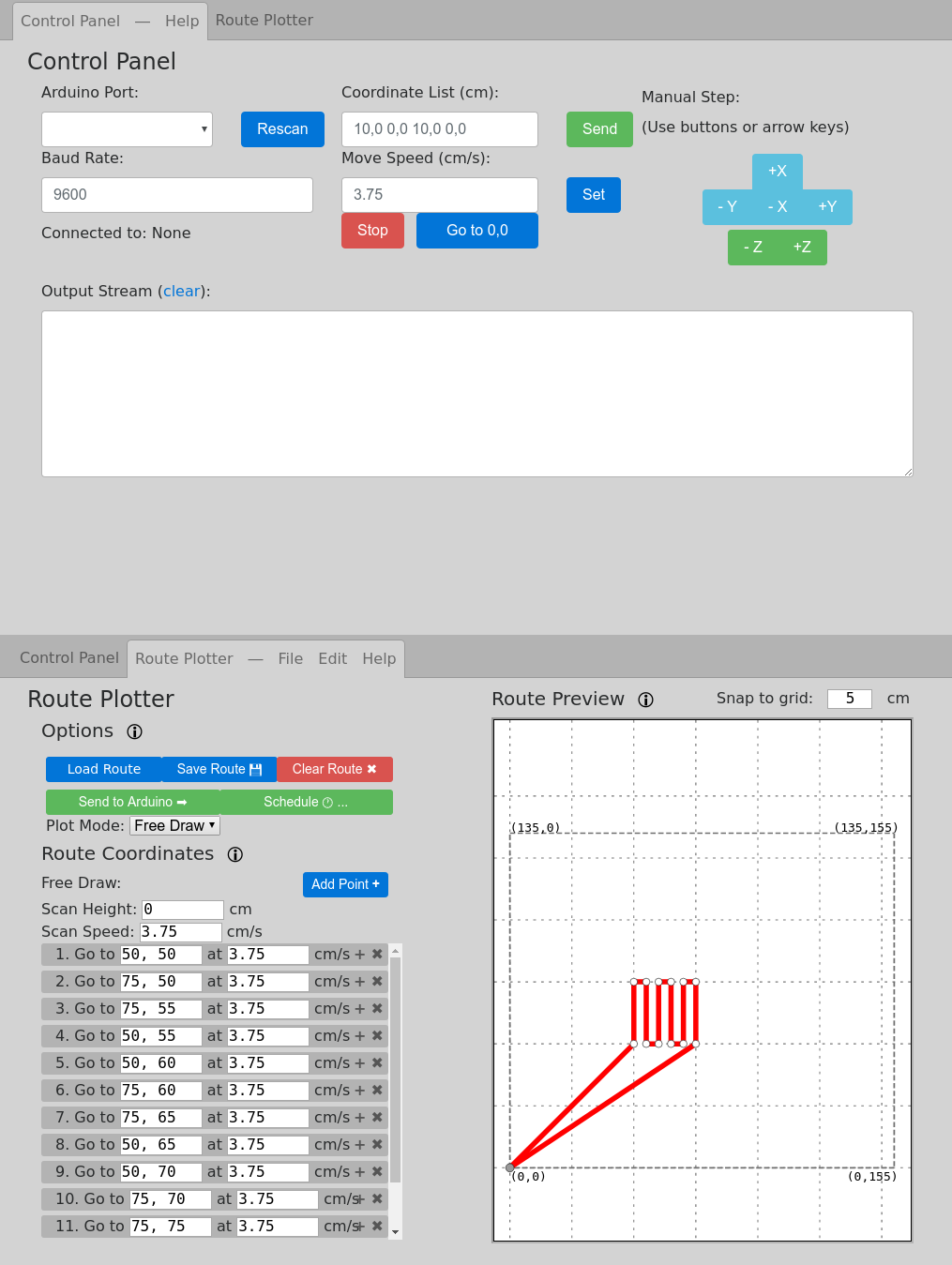

Supplement: Supplementary file 6 — Additional file 6. Screenshots of Ardupy’s manual control and route planner menus. Users can control HyperScanner with manual control or through creating paths by click-and-dragging waypoints on the map panel. [file 13007_2019_392_MOESM6_ESM.tif]

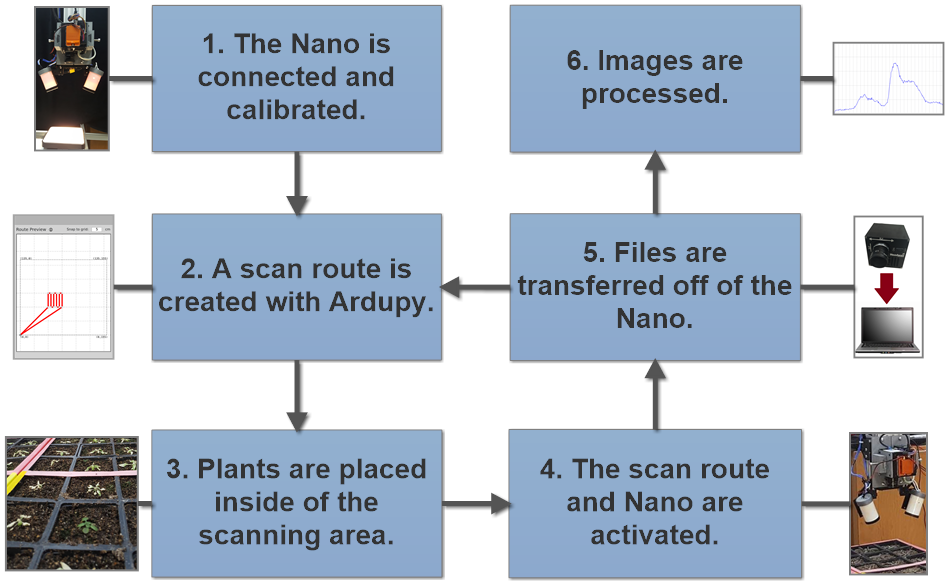

Supplement: Supplementary file 7 — Additional file 7. A flowchart describing HyperScanner’s operational procedure. [file 13007_2019_392_MOESM7_ESM.tif]
